# Effects of Two-Phase Treatment with the Herbst and Preadjusted Edgewise Appliances on the Upper Airway Dimensions

**DOI:** 10.1155/2016/4697467

**Published:** 2016-03-17

**Authors:** Woei Li Koay, Yanqi Yang, Christine Shuk Kwan Tse, Min Gu

**Affiliations:** Orthodontics, Faculty of Dentistry, The University of Hong Kong, 34 Hospital Road, Sai Ying Pun, Hong Kong

## Abstract

*Aims*. To assess the effects of two-phase treatment with the Herbst and the preadjusted edgewise appliances on upper airway dimensions and to investigate the correlation between changes in the upper airway dimensions and skeletal morphologies.* Methods*. A total of 27 Chinese male adolescents aged 12.8 ± 1.3 years were selected. Lateral cephalograms were collected to assess the skeletal morphology and upper airway dimensions.* Results*. Following Herbst appliance treatment, the upper airway space was significantly enlarged, with the retropalatal (U-MPW) increasing by 1.1 ± 1.6 mm (*P* < 0.001), the retroglossal (PASmin) increasing by 1.3 ± 2.9 mm (*P* < 0.05), and the hypopharynx (V-LPW) enlarging by 1.6 ± 3.0 mm (*P* < 0.01). PASmin was found to show a negative correlation to the mandibular plane angle (MnPl-SN) by *r* = −0.413 (*P* < 0.05). There was no significant change (*P* > 0.05) in upper airway dimensions during the second-phase treatment.* Conclusions*. Herbst appliance treatment increased the oropharyngeal and hypopharyngeal airway dimensions among adolescents with Class II malocclusion, and the effects were maintained throughout the second treatment phase with a preadjusted edgewise appliance. There was a negative correlation between the change in the depth of the retroglossal pharynx and the mandibular plane angle.

## 1. Introduction

Mandible advancement devices (MADs) are known to improve the airway space, particularly in adult patients with sleep apnoea and snoring problems [1–3]. MADs are nonsurgical approaches and are currently recognised as the primary noncontinuous positive airway pressure (CPAP) therapy [1] used to treat mild to moderate obstructive sleep apnoea (OSA) and daytime sleepiness, and for those who do not tolerate CPAP [2, 4]. MADs are effective in patients over 65 years old with good dental health [5]. They advance the mandible forward by holding the teeth to increase the patency of the airway space and thus reduce snoring, improve the quality of sleep, and reduce daytime sleepiness. There is no single type of MAD that influences perceived treatment efficacy above others, but the majority of studies have shown improved subjective outcomes with MAD. This suggests that mandibular advancement is a crucial design feature of oral appliance therapy for obstructive sleep apnoea syndrome [6–9].

Functional appliances have similar effects to MADs, also advancing the mandible to a forward position [10, 11]. They are commonly used among skeletal Class II children with retrognathic mandibles [12, 13]. When the muscles attached to the mandible are stretched, these appliances have been shown to help reposition the mandible by remodelling the condyle, and the force generated is sufficient to induce secondary growth at the condylar head, therefore changing the growth pattern of the face [14, 15]. Functional appliances are also believed to affect airway correction by preventing the tongue from dropping backward and blocking the airway when the mandible is held in a forward position [16]. TheHerbst appliance is a fixed functional appliance that is cemented temporarily onto the teeth without relying on patient compliance and may help with pharyngeal space correction [11, 16].

Approximately 2–10% of school-aged children suffer from sleep-disordered breathing (SDB) [17], and constriction of the pharyngeal airway space is common in these patients [18]. Daytime sleepiness is also related to SDB, which is potentially associated with certain Class II tendency malocclusions, such as an increased overjet and bilateral Class II molar relationship [19]. Therefore, an early diagnosis is recommended for children who potentially have this sleep problem, enabling them to receive proper intervention.

The aims of this study are to assess the effects of the Herbst and the preadjusted edgewise appliances on upper airway dimensions among adolescents with Class II malocclusion and to investigate the correlation between changes in the upper airway dimensions and the skeletal morphology after two-phase treatment with these appliances.

## 2. Materials and Methods

This study was approved by the Institutional Review Board of the University of Hong Kong/Hospital Authority, Hong Kong West Cluster (IRB Reference number: UW 12-405).

### 2.1. Sample Size Calculation

Calculation of the sample size was based on the ability to detect a clinically relevant difference in the changes of the depth of the retroglossal pharyngeal airway space (PASmin) by 1.87 mm after the functional appliance treatment [13]. On this basis, with an alpha of 0.05 and a study power of 0.80, 26 subjects were required for the study group.

### 2.2. Subjects

A sample of Chinese male subjects who underwent orthodontic treatment at the Prince Philip Dental Hospital from 1999 to 2010 was recruited.

The inclusion criteria were 1, male; 2, in the age range of 11 to 14 at pretreatment (*T*0); 3, no permanent teeth extracted; 4, having received two-phase treatment with a Herbst appliance and a preadjusted edgewise appliance; 5, complete lateral cephalograms obtained at pretreatment (*T*0) and immediate post-Herbst (*T*1) and postedgewise treatment (*T*2). The exclusion criteria for the study were patients with craniofacial syndromes and cleft lips or palates.

Based on the selection criteria, consecutive cases that had undergone orthodontic treatment at the Prince Philip Dental Hospital from 1999 to 2010 were screened, and 27 male patients were selected for the study. The mean average age of the subjects who began the Herbst treatment was 12.8 ± 1.3 years. They completed phase 1 treatment by the mean age of 13.9 ± 1.5 years, which was followed by the preadjusted edgewise treatment. The mean of the two-phase treatment time duration was 3.0 ± 1.1 years. The same sample was used in a previous study [20] to evaluate the change of mandibular position.

### 2.3. Cephalometric Analysis

**Figure 1:**
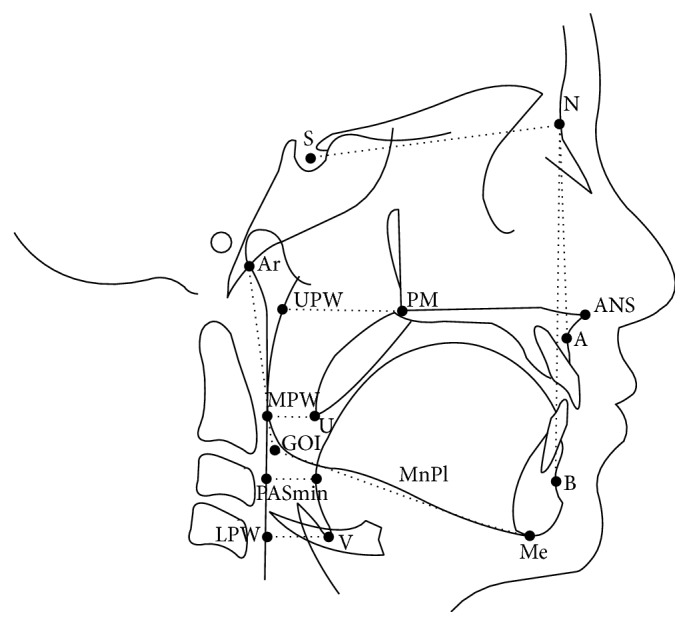
Landmarks and measurements of the upper airway and skeletal morphology.

Lateral cephalometric radiographs were collected and analysed using CASSOS software (Soft Enable Technology Limited, Hong Kong, China). After calibration, the landmarks were identified by the same examiner (KWL), and the parameters for the skeletal morphology and upper airway dimensions were obtained. Landmarks [21] representing the four parts of the upper airway, namely, the nasopharyngeal (PM-UPW), retropalatal (U-MPW), retroglossal (PASmin), and hypopharyngeal (V-LPW), were used to assess the dimension changes of the upper airway. Skeletal morphology was represented by four different parameters, SNA, SNB, ANB, and mandibular angle (MnPl/SN), to assess the sagittal and vertical changes of the skeletal pattern (Table 1, Figure 1).

Pretreatment (*T*0), post-Herbst treatment (*T*1), and postedgewise treatment (*T*2) lateral cephalograms were analysed, and the changes in the depth of the four upper airway spaces (PM-UPW, U-MPW, PASmin, and V-LPW) and skeletal morphology (ANB angle, SNA angle, SNB angle, and MnPl/SN angle) were used to determine the skeletal and pharyngeal space response to treatment with a functional appliance followed by a preadjusted edgewise appliance (Table 3).

Comparisons were made between patients in the same group using different time points at pretreatment (*T*0), post-Herbst treatment (*T*1), and postedgewise treatment (*T*2) to assess the changes. Those due to functional appliance therapy from the pretreatment stage to the post-Herbst stage were measured as (*T*1 − *T*0) between the two time points. The mean difference change found in the postedgewise stage was then compared (*T*2 − *T*1) to assess the changes brought by the second stage treatment. The overall treatment effects (*T*2 − *T*0) were obtained by comparing the pretreatment stage and the end treatment of the preadjusted edgewise appliance (Table 3).

### 2.4. Method Errors

Error studies were performed on 25 randomly selected lateral cephalograms, and the measurements were repeated after a two-week time interval by the same examiner (KWL). Error studies were carried out using Dalberg's formula and a paired *t*-test (Table 2). Dalberg's formula [22] was as follows: $ME=\sqrt{\Sigma d^{2}/2n}$, where ME is the method error, *d* is the difference between the first and the second measurements, and *n* is the number of repeated measurements. The difference between the repeated measurements was not statistically significant in the paired *t*-test and did not exceed 1 mm for the linear and 1° for the angular measurements (Table 2), which were acceptable levels of error.

### 2.5. Statistical Analysis

The normality of the data appeared to be valid (Shapiro-Wilk test). Comparative statistical analysis of the data was carried out using a paired *t*-test to analyse the significant differences between the changes during the treatment periods (*T*1 − *T*0, *T*2 − *T*1, and *T*2 − *T*0) (Table 3). The probability value *P* < 0.05 was considered significant, with *P* < 0.01 and *P* < 0.001 considered highly significant. The correlation between the changes in the pharyngeal space and the skeletal morphology was analysed using the Spearman rank correlation, and the statistical significance was set at the level of *P* < 0.05 (Table 4). SPSS software (IBM SPSS Statistics version 20, IBM Corp.) was used to carry out the statistical analyses.

## 3. Results

### 3.1. Changes in Skeletal Morphology

The paired *t*-test revealed a statistical increase in SNB of 2.4 ± 2.0 degrees (*P* < 0.001) and ANB was reduced by 1.7 ± 2.2 degrees (*P* < 0.001) after the Herbst treatment (*T*1 − *T*0). The changes in SNA and the MnPl/SN angle were not statistically significant. None of the variables showed a statistically significant change during the second phase of the preadjusted edgewise appliance treatment (*T*2 − *T*1). However, the data showed a similar change pattern at the completion of the two-phase treatment (*T*2 − *T*0), which was 1.8 ± 2.4 degrees (*P* < 0.001) and −2.0 ± 2.0 degrees (*P* < 0.001), respectively (Table 3).

### 3.2. Changes in the Upper Airway Dimensions

The upper airway space was significantly enlarged by a similar amount after the Herbst treatment (*T*1 − *T*0) (Table 3). The retropalatal region (U-MPW) increased by 1.1 ± 1.6 mm (*P* < 0.001), the retroglossal region (PASmin) by 1.3 ± 2.9 mm (*P* < 0.05), and the hypopharynx region (V-LPW) by 1.6 ± 3.0 mm (*P* < 0.01). There was an insignificant reduction in the nasopharynx region (PM-UPW) (*P* > 0.05). The improvement in the upper airway dimensions was maintained throughout the second-phase treatment with the preadjusted edgewise appliance (*T*2 − *T*1). The overall therapeutic effect (*T*2 − *T*0) was that V-LPW had the highest dimensional changes, of 2.7 ± 2.6 mm (*P* < 0.001), followed by the increase of PASmin by 1.4 ± 2.8 mm (*P* < 0.05) and U-MPW by 0.9 ± 2.1 mm (*P* < 0.05) (Table 3).

### 3.3. Correlations between the Changes in the Skeletal Morphology and Upper Airway Dimensions

In the main, the changes in both the skeletal morphology and airway dimensions occurred in the Herbst treatment phase, so changes in the first phase (*T*1 − *T*0) and also those after the two-stage treatment (*T*2 − *T*0) was completed were further analysed to investigate the correlation between the skeletal morphology and upper airway dimensions (Table 4).

There was no correlation between the changes in the upper airway dimensions and the sagittal skeletal morphology after the two-phase treatment (*T*2 − *T*0) (Table 4) in the analysis. However, PASmin showed a negative correlation to the mandibular plane angle (MnPl/SN) by *r* = −0.413 (*P* < 0.05) after the Herbst treatment (*T*1 − *T*0) (Table 4).

## 4. Discussion

### 4.1. The Influence of the Herbst on the Upper Airway Dimensions

Our study showed that, during the Herbst appliance treatment, SNB was increased; the airway dimensions were effectively increased in similar amount from the medium pharyngeal space of the oropharynx to the hypopharynx, which suggested that the Herbst effectively and consistently influenced most areas of the upper airway during its active phase. The result was found to be in line with previous studies [10]. Schütz et al. [10] showed that the Herbst improved nocturnal breathing in the short term by increasing the airway space associated with the correction of mandibular retrognathism. Other studies also reported on the effectiveness of different type of functional appliances on the increase of pharyngeal space in long term follow-up [23, 24].

The Herbst appliance is considered an effective functional appliance for altering airway dimensions due to its full-time action and the fact that it does not rely on patient compliance [25]. The reduction of SNA by 0.8 degrees in postedgewise stage (*T*2 − *T*1) was due to the remodelling of the anterior alveolar bone after retraction of upper labial segment especially in extraction cases for the dental camouflage of the Class II skeletal pattern [26]. The small reduction of SNA was not statistically significant to affect the distance between the soft palate and the posterior pharyngeal wall.

The overall treatment effect on the upper airway dimensions in the postedgewise stage (*T*2 − *T*0) was increased and the greatest dimensional change was found to be at the lowest part of the airway level which was the hypopharynx region. This may be due to some treatment plus growth effect although the change was minimal and not significant. However, it is unethical to have an untreated control group to distinguish between the appliance effect and growth effect with the age match.

It is well known that the different genders will reach their peak growth period at different times [21, 23, 27]. Males showed significantly greater height growth and increase in pharyngeal length although there are no significant changes between genders in long term follow-up [23]. Therefore, we attempted to minimise the variability of our results due to this different growth factor by selecting only Hong Kong Chinese boys as our study subjects.

### 4.2. Correlations between the Changes of the Skeletal Morphology and the Changes of the Upper Airway Dimension

The present study also found a significant correlation (*P* < 0.05), although weak (*r* = −0.413), between the changes in the airway dimensions and the vertical changes in the mandibular plane angle. The change in the retroglossal pharyngeal space (PASmin) was inversely correlated with the mandibular plane angle (MnPl/SN) after the first phase of treatment with the Herbst appliance (*T*1 − *T*0). That is, when the mandibular was advanced during the Herbst treatment, there were an increase at the medium pharyngeal airway and a reduction in the mandibular plane angle. There was similar finding by Hänggi et al. [23] of weak correlation (*r* = −0.22, *P* = 0.22) between the changes of pharyngeal space and the angular changes of MnPl/SN when the growth was taken into consideration. However, Han et al. [24] showed a significant interaction effect between the upper oropharyngeal region, gonial angle, and mandibular plane angle (*P* < 0.05). Therefore, it is important to obtain good vertical control of the skeletal pattern, which could benefit airway modification, particularly when treating skeletal CII cases.

### 4.3. Lateral Cephalometric Analysis and Alternative Procedures

Lateral cephalogram records can only provide a two-dimensional assessment, and computed tomography (CT) [28] or magnetic resonance imaging (MRI) will be more appropriate to give a three-dimensional (volume) assessment of the airway space.

The measurements acquired from both modalities (lateral cephalogram and CT) are reliable and reproducible, but CT gives a better assessment of the cross-sectional dimensions of the airway space [28]. However, proper justification is crucial before exposing patients to the higher radiation of CT investigation, which may not result in significant changes in the treatment modalities.

In this study, we were not able to relate our findings to the sleep quality improvement of the patients. The patients received treatment to correct the retrognathic mandible and did not complain of airway problems nor did they undergo polysomnography (PSG). Retrospectively, we note that there was no special instruction given to the patient to locate the tongue or determine the actual tongue position in the lateral cephalogram records, which could affect the findings of our study.

Due to ethical limitation, this study did not include a control group with untreated Class II malocclusion. The changes following treatment should be seen as combination effects of growth and treatment.

## 5. Conclusions

The oropharyngeal and hypopharyngeal airway dimensions were increased among adolescents presenting with Class II malocclusion during Herbst appliance treatment, and the effects were maintained throughout the second treatment phase with a preadjusted edgewise appliance. There was a negative correlation between the change in the depth of the retroglossal pharynx and the mandibular plane angle.

## Figures and Tables

**Table 1:** Definition of cephalometric landmarks and measurements of Figure 1.

	Definition
Landmarks	
ANS	Anterior nasal spine, the tip of the median, sharp bony process of the maxilla
PM	Pterygomaxillary, the intersection between the nasal floor and the posterior contour of the maxilla [29]
UPW	Upper pharyngeal wall, point of intersection of the line NL to the posterior pharyngeal wall
MPW	Middle pharyngeal wall, intersection of the perpendicular line from U to the posterior pharyngeal wall
LPW	Lower pharyngeal wall, intersection of the perpendicular line from V to the posterior pharyngeal wall
U	Uvula, the tip of the uvula
V	Vallecula, the intersection of the epiglottis and the base of the tongue
S	Center of the sella turcica
N	Nasion, the deepest point in the concavity of nasofrontal suture
A	A point, the deepest point in the concavity of the anterior maxilla between the anterior nasal spine and the alveolar crest
B	B point, the deepest point in the concavity of the anterior mandible between the alveolar crest and the pogonion
Gn	Gnathion, the most anteroinferior point on the bony chin
Me	Mention, the most inferior point on the body chin
Ar	The intersection of the posterior border of the ramus with inferior surface of the cranial base
GOI	Gonial intersection, the intersection of the mandibular plane with a plane through Ar, posterior and along the portion of the mandibular ramus inferior to it [30]
NL	Nasal line, line joining the ANS and PM
MnPl	Mandibular plane, line joining Me and GOI
Measurements	
PM-UPW (mm)	Depth of the nasopharyngeal airway space from PM to UPW
U-MPW (mm)	Depth of the oropharyngeal airway space from U to MPW
PASmin (mm)	Shortest distance between the base of the tongue and the posterior pharyngeal wall, the narrowest sagittal airway space
V-LPW (mm)	Depth of the hypopharyngeal airway space from V to LPW
SNA (°)	Angle between the S-N line and the N-A line
SNB (°)	Angle between the S-N line and the N-B line
ANB (°)	Angle between the N-A line and the N-B line
MnPl/SN (°)	Mandibular plane angle, the angle between the MnPl and the S-N line

**Table 2:** Method error analyses of landmark input using Dahlberg's formula and paired *t*-test.

Measurements	Dahlberg's formula	*t*-test *P* value
Depth of upper airway (mm)		
PM-UPW	0.981	0.233
U-MPW	0.448	0.180
PASmin	0.362	0.301
V-LPW	0.680	0.084

Skeletal morphology (°)		
SNA	0.506	0.072
SNB	0.356	0.088
ANB	0.497	0.561
MP-SN	0.847	0.287

**Table 3:** Descriptive statistics and comparison of variables at pretreatment (*T*0), post-Herbst treatment (*T*1), and postedgewise treatment (*T*2) periods (*n* = 27).

	*T*0		*T*1		*T*2		*T*1 − *T*0		*P* value	*T*2 − *T*1		*P* value	*T*2 − *T*0		*P* value
	Mean	SD	Mean	SD	Mean	SD	Mean	SD		Mean	SD		Mean	SD	
Age (years)	12.8	1.3	13.9	1.5	15.7	1.8	1.1	0.3		1.9	1.1		3.0	1.1	

Depth of upper airway (mm)															
PM-UPW	25.5	3.8	25.2	4.7	25.4	3.6	−0.3	4.5	0.754	0.1	3.5	0.830	−0.1	3.6	0.852
U-MPW	10.3	4.1	11.5	2.9	11.2	2.5	1.1	1.6	0.001^*∗∗*^	−0.3	2.5	0.572	0.9	2.1	0.043^*∗*^
PASmin	10.6	1.5	11.9	3.5	12.0	3.3	1.3	2.9	0.029^*∗*^	0.1	3.3	0.886	1.4	2.8	0.016^*∗*^
V-LPW	17.6	7.8	19.2	3.9	20.3	3.5	1.6	3.0	0.008^*∗∗*^	1.1	2.9	0.069	2.7	2.6	<0.001^*∗∗∗*^

Skeletal morphology (°)															
SNA	81.3	4.0	82.0	3.8	81.2	3.3	0.7	2.2	0.115	−0.8	2.9	0.148	−0.1	2.7	0.820
SNB	74.6	2.9	77.0	3.9	76.5	4.1	2.4	2.0	<0.001^*∗∗∗*^	−0.6	2.0	0.153	1.8	2.4	<0.001^*∗∗∗*^
ANB	6.7	3.3	5.0	2.3	4.8	2.1	−1.7	2.2	<0.001^*∗∗∗*^	−0.3	2.0	0.506	−2.0	2.0	<0.001^*∗∗∗*^
MnPl-SN	35.5	3.3	34.6	7.3	35.0	8.1	−0.8	2.1	0.052	0.4	3.0	0.553	−0.5	2.7	0.357

^*∗*^ *P* < 0.05; ^*∗∗*^ *P* < 0.01; ^*∗∗∗*^ *P* < 0.001.

**Table 4:** Results of Spearman's correlation analysis for changes of skeletal morphology and dimension of upper airway.

		Depth of upper airway (*T*1 − *T*0)				Depth of upper airway (*T*2 − *T*0)			
		PM-UPW	U-MPW	PASmin	V-LPW	PM-UPW	U-MPW	PASmin	V-LPW
Skeletal morphology (*T*1 − *T*0)									
SNA	Coefficient (*r*)	0.229	0.034	0.309	0.146	−0.232	−0.295	0.004	0.324
	*P* value	0.251	0.867	0.116	0.469	0.244	0.136	0.984	0.099
SNB	Coefficient (*r*)	−0.161	0.100	0.296	0.196	−2.340	−0.009	−0.062	0.303
	*P* value	0.422	0.621	0.134	0.326	0.244	0.964	0.757	0.125
ANB	Coefficient (*r*)	0.341	0.002	0.071	0.007	−0.102	−0.319	0.015	0.046
	*P* value	0.082	0.993	0.725	0.974	0.613	0.105	0.941	0.820
MnPl-SN	Coefficient (*r*)	0.136	−0.127	−0.413	−0.150	−0.052	−0.116	−0.280	−0.353
	*P* value	0.500	0.526	0.032^*∗*^	0.454	0.797	0.563	0.157	0.071

^*∗*^ *P* < 0.05.
